# Iron supplementation switches mode of cell death to ferroptosis during acetaminophen-induced liver injury in mice rendering it resistant to N-acetylcysteine

**DOI:** 10.1016/j.tox.2025.154221

**Published:** 2025-06-13

**Authors:** Olamide B. Adelusi, Aparna Venkatraman, Jephte Y. Akakpo, Anup Ramachandran, Hartmut Jaeschke

**Affiliations:** a Department of Pharmacology, Toxicology & Therapeutics, University of Kansas Medical Center, Kansas City, KS, USA; b Division of Neonatology, Department of Pediatrics, Children’s Mercy Kansas City, Kansas City, MO, USA

**Keywords:** drug hepatotoxicity, N-acetylcysteine, ferroptosis, lipid peroxidation, peroxynitrite, 4-methylpyrazole

## Abstract

Acetaminophen (APAP) overdose can cause liver injury and is the leading cause of acute liver failure in Western countries. Hepatocellular necrosis induced by APAP involves the formation of a reactive metabolite, triggering mitochondrial oxidant stress and peroxynitrite formation. Iron-catalyzed protein nitration is critical for mitochondrial dysfunction and cell death in the absence of lipid peroxidation (LPO). However, co-treatment of APAP and ferrous sulfate aggravated protein nitration and liver injury but also triggered extensive LPO (measured as malondialdehyde and hydroxy eicosatetraenoic acid (HETE) species). The objective of this study was to evaluate whether the aggravated injury under these conditions is caused by a combination of protein nitration and LPO or if LPO is now the dominant injury mechanism. To test this, C57BL/6 J mice were co-treated with APAP (300 mg/kg) and a moderate dose of ferrous sulfate (0.15 mmol/kg) for 6 h. Some animals also received a dose of Mito-TEMPO, the mitochondria-targeted SOD mimetic, or minocycline, an inhibitor of mitochondrial iron uptake. Although Mito-TEMPO and minocycline eliminated protein nitration and liver injury after APAP alone, these interventions did not affect LPO and only had a moderate effect on protein nitration and liver injury in the APAP+Fe^2+^ group, suggesting LPO as the main mechanism of cell death. Consistent with these findings, delayed treatment with clinically relevant antidotes N-acetylcysteine and fomepizole did not reduce LPO or liver injury. Thus, liver injury after APAP+Fe^2+^ is no longer primarily driven by mitochondrial oxidant stress and peroxynitrite-mediated necrosis but by lipid peroxidation and a ferroptosis-like cell death.

## 1. Introduction

Acetaminophen (APAP) is an effective analgesic and antipyretic, and one of the most consumed drugs worldwide. Safe at therapeutic doses of ≤ 4 g/day in most adults, an overdose can cause liver injury, and under extreme circumstances, acute liver failure and death (Larson, 2007; Stravitz and Lee, 2019). In Western countries, APAP overdose is the dominant etiology of acute liver failure (Bernal and Wendon, 2013). The mouse model of APAP toxicity faithfully replicates the human pathophysiology (Jaeschke et al., 2014; Ramachandran and Jaeschke, 2017). During investigation with this model, due to the clinical importance of APAP overdose, several key mechanisms that initiate and propagate signaling events in hepatocytes have been identified. These include the formation of a reactive metabolite N-acetyl-p-benzoquinone imine (NAPQI) by the cytochrome P450 enzyme 2E1 (Cyp2E1), hepatic glutathione (GSH) depletion, and protein adducts formation, especially on mitochondria. The subsequent initiation of a mitochondrial oxidant stress and its amplification by c-jun N-terminal kinase (JNK) activation leads to opening of the mitochondrial permeability transition pores (MPTP) with release of intermembrane proteins such as endonuclease G and apoptosis inducing factor (AIF), which translocate to the nucleus and trigger DNA fragmentation (Jaeschke and Ramachandran, 2024a,b; Ramachandran and Jaeschke, 2019). These signaling events cause hepatocellular necrosis characterized morphologically by cell and organelle swelling, karyorrhexis, and cell contents release (Gujral et al., 2002).

The most controversial topic was and still is the role of reactive oxygen species (ROS) in the pathophysiology of APAP hepatotoxicity. Early studies proposed superoxide formation by cytochrome P450 enzymes during metabolism of APAP and the resulting lipid peroxidation (LPO) as mechanism of cell death (Wendel and Feuerstein, 1981; Wendel et al., 1979, 1982). However, no direct evidence for an oxidant stress was observed during APAP metabolism in vivo (Lauterburg et al., 1984; Smith and Jaeschke, 1989) and in isolated hepatocytes (Bajt et al., 2004). In addition, LPO as a cell death mechanism was also questioned due to the quantitatively very limited LPO and the fact that excess vitamin E did not protect (Adelusi et al., 2022; Knight et al., 2003; Siegers et al., 1988; Younes and Siegers, 1985). However, an oxidant stress in mitochondria was identified (Jaeschke, 1990; Knight et al., 2001), which is derived from complex I and III of the electron transport chain (Nguyen et al., 2021). Despite this insight, the mechanism of ROS involvement in APAP-induced cell death remained unclear.

Progress was made when evidence for peroxynitrite formation in centrilobular hepatocytes was discovered after an APAP overdose (Hinson et al., 1998). Peroxynitrite is a potent oxidant and nitrating species, which is formed when the anion radical superoxide spontaneously reacts with a nitric oxide radical (Prolo et al., 2024). Because superoxide is mainly released into the mitochondrial matrix, peroxynitrite is formed in the mitochondria, where it causes oxidative damage to mitochondrial DNA and extensive nitration of mitochondrial proteins (Cover et al., 2005). The critical importance of peroxynitrite in APAP-induced cell death was further supported by the protection during accelerated restoration of hepatic and mitochondrial GSH levels that scavenged peroxynitrite (James et al., 2003; Knight et al., 2002; Saito et al., 2010), the protection with mitochondria-targeted SOD mimetics, which prevent peroxynitrite formation (Du et al., 2017; He et al., 2023) and the aggravation of the injury with MnSOD deficiency that caused enhanced peroxynitrite formation (Ramachandran et al., 2011). Questions again came up when it was shown that lysosomal ferrous iron is taken up into mitochondria and facilitates the MPTP opening (Kon et al., 2010; Hu et al., 2016). However, it is known that protein nitration requires metal ion catalysis (Ferrer-Sueta and Radi, 2009). Thus, iron chelation protects against APAP toxicity by elimination of nitration in the absence of LPO under normal conditions (Adelusi et al., 2022). However, when animals were pretreated with ferrous iron, APAP-induced liver injury was aggravated with increased protein nitration and dramatically increased LPO (Adelusi et al., 2022, 2024). Because the dominant mechanism of the enhanced cell death in the context of iron supplementation was unclear, the objective of the current study was to assess whether iron supplementation will shift the mechanism of injury predominantly to LPO, i.e., a ferroptosis-like cell death, or if protein nitration remains the critical event in the pathophysiology.

## 2. Materials and methods

### 2.1. Animals

8–10-week-old male C57BL/6J mice obtained from Jackson Laboratories (Bar Harbor, ME) were used in all experiments. The mice were kept in an environmentally controlled room and allowed to acclimatize for several days before the experiments. Mice were kept under a 12-hour light/dark cycle and provided food and water *ad libitum*. All experiments were carried out in accordance with the National Research Council’s Guide for the Care and Use of Laboratory Animals. All experiments were approved by the Institutional Animal Care and Use Committee of the University of Kansas Medical Center. All procedures reported in the study involving animals complied with the ARRIVE guidelines.

### 2.2. Experimental design

All chemicals and reagents were obtained from Sigma-Aldrich (St. Louis, MO) unless mentioned otherwise. Overnight fasted mice received intraperitoneal injections of APAP (300 mg/kg) with or without co-treatment with 0.15 mmol/kg FeSO_4_, which represents a non-toxic dose of iron (Gibson et al., 1996). In addition to APAP with or without FeSO_4_, groups of mice received the following treatments intraperitoneally: minocycline (10 mg/kg) 1 h before APAP, Mito-TEMPO (20 mg/kg) 90 min after APAP, N-acetylcysteine (500 mg/kg) 90 min after APAP, or fomepizole (4-MP) (50 mg/kg) 90 min after APAP. Mice were sacrificed 6 h after APAP under isoflurane anesthesia. Blood was collected from the inferior vena cava into heparin coated syringes and livers were harvested. To obtain plasma, blood was centrifuged at 20, 000 g for 3 min. Livers were dissected into sections which were snap frozen in liquid nitrogen, fixed overnight in 10 % phosphate buffered formalin, or placed in a sucrose/mannitol buffer for subcellular fractionation.

### 2.3. Biochemical assays

Plasma alanine amino transferase (ALT) activity was measured using an ALT test kit (Pointe Scientific, Canton, MI) according to the manufacturer’s instructions. Hepatic malondialdehyde (MDA) was measured in freshly homogenized liver tissue as described (Adelusi et al., 2022).

### 2.4. Histology

Livers were fixed by overnight immersion in 10 % phosphate buffered formalin before being embedded in paraffin and cut into 5-micron thick sections. Tissue sections were deparaffinized and rehydrated followed by hematoxylin and eosin (H&E) staining to visualize the necrotic areas. Images were acquired on a Nikon Eclipse Ti2 microscope. Necrotic areas were quantified on three randomly selected 100X images per animal using ImageJ (Schindelin et al., 2012).

### 2.5. TUNEL assay

The TUNEL (terminal deoxynucleotidyl transferase dUTP nick-end labeling) assay was performed on deparaffinized and rehydrated liver sections using the In Situ Cell Death Detection Kit (Roche, #11684809910) according to the manufacturer’s instructions.

### 2.6. Immunofluorescence

5-micron thick liver tissue sections were deparaffinized, rehydrated, and subjected to antigen retrieval using citrate buffer (Sigma Aldrich) for 20 min at > 95°C in a steamer. Sections were permeabilized with 1x PBS + 0.05 % Tween-20 (1x PBS-T) and blocked with Power Block Universal Blocking Reagent (BioGenex) for 1 h at room temperature. The primary antibody was diluted in antibody diluent reagent (Invitrogen). Sections were then incubated overnight at 4°C with the primary antibody (3-Nitrotyrosine, Mouse, 1:75, Abcam Cat. # ab61392). After three washes with 1x PBS-T, the tissue sections were stained with the secondary antibody conjugated to Alexa Fluor 488 (1:100, Life Technologies) and DAPI (1:1000, Sigma) for 1 h at room temperature in the dark. Slides were mounted using Fluorogel with Tris buffer (Electron Microscopy Sciences). Negative controls were obtained for slides of all treated groups using the same procedure without the primary 3-nitrotyrosine antibody. Images were captured on a Nikon Eclipse Ti2 epifluorescence microscope using a Plan-Apochromat 20x/0.8 air objective. Images were processed by channel using Fiji/ImageJ. Fluorescence intensity measurements were performed in Fiji (ImageJ) as described earlier (Shihan et al., 2021).

### 2.7. Subcellular fractionation and Western blotting

Subcellular fractions were isolated by homogenization of freshly collected liver tissue in ice cold mannitol/sucrose buffer, followed by differential centrifugation as described in detail (Adelusi et al., 2022). The BCA assay (Thermofisher Scientific, Waltham, Massachusetts) was performed on subcellular fractions and whole liver homogenate to determine protein concentration before performing Western blotting as described (Adelusi et al., 2024). The primary antibodies used are as follows: rabbit anti-AIF (1:1000, CST, #4642S), mouse anti-Cytochrome C (1:500, Santa Cruz, sc-13156), rabbit anti-GAPDH (1:1000, CST, #9252), rabbit anti-p-JNK (1:1000, CST, #9251), and rabbit anti-JNK (1:1000, CST, #9252). The following secondary antibodies were used: anti-rabbit IgG, HRP-linked antibody (1:5000, CST, #7074) and anti-mouse IgG, HRP-linked antibody (1:5000, CST, #7076).

### 2.8. Analysis of hydroxy eicosatetraenoic acids (HETEs)

#### 2.8.1. Liver tissue sample preparation for mass spectrometry

The methanol extraction process was conducted by weighing approximately 10 mg of tissue and adding it to 90 μL of methanol, 5 μL of 10 mg/mL butylated hydroxytoluene dissolved in ethanol, and 5 μL of internal standard solution of 12 HETE D8 (400 ng/mL) in a disposable microcentrifuge tube (Liakh et al., 2019). If a higher tissue mass was required, the volume of methanol was proportionally increased to maintain the tissue-to-methanol ratio at 1:9. The mixture was homogenized thoroughly, vortexed for 5 min, and centrifuged at 10,000 ×g for 15 min at 4 °C. Following centrifugation, the supernatant was carefully collected and subjected to evaporation at 55 °C under a pressure of 16 psi to concentrate the sample and enhance detection sensitivity. The resulting pellet was resuspended in 60 μL of 50 % methanol and transferred into silanized micro inserts. To precipitate additional proteins, the samples were stored at −80 °C for a minimum of 10 min. After warming the samples back to room temperature, if further precipitate formed, they were centrifuged again at 1000 ×g for 10 min. The supernatant from this step was then transferred into new silanized micro inserts for subsequent analysis.

#### 2.8.2. Ultra performance liquid chromatography conditions

UPLC separation of HETEs (Cayman, Ann Arbor, MI) was conducted using an ACQUITY UPLC I-Class System equipped with an ACQUITY Premier BEH C18 analytical column (2.1 ×150 mm), which was maintained at a temperature of 45 °C. A sample aliquot of 5 μL was injected onto the column and eluted under reversed-phase gradient conditions (Brose et al., 2013). The mobile phase consisted of two components: mobile phase A, which contained 0.01 % formic acid in water (aqueous), and mobile phase B, which comprised 0.01 % formic acid in a mixture of acetonitrile and methanol at a ratio of 90:10. Separation of HETEs species was achieved through a linear gradient program. The gradient began with an initial composition of 2 % mobile phase B, increasing to 98 % over 7 min. This was followed by a 4-minute column flush step to ensure complete elution of retained analytes, after which the system underwent re-equilibration to restore the starting conditions for subsequent runs.

#### 2.8.3. Mass spectrometer conditions

HETEs species were detected and analysed using Multiple Reaction Monitoring (MRM) on a Xevo TQ-XS Mass Spectrometer (Waters Corporation). All experiments were conducted in negative electrospray ionization (ESI-) mode. The capillary voltage was 2.5 kV, while the cone was 20 V, and ion source temperature was maintained at 150 °C. Additionally, the cone gas flow rate was fixed at 150 L/hr, and the desolvation temperature was adjusted to 1000 °C. The following MRM transitions were used: 5 HETE (319.21–114.99), 15 HETE (319.21–219.16), 12 HETE (319.21–179.1), and 12 HETE D8 (327.28–184.11).

### 2.9. Statistical analysis

One-way analysis of variance (ANOVA) followed by a post-hoc Bonferroni test was used to make comparisons between groups. All experiments had an n = 4–6 biological replicates. P values < 0.05 were taken to be statistically significant. Statistical analysis was performed on GraphPad Prism version 8.0.1 for Windows (GraphPad Software, San Diego, California).

## 3. Results

### 3.1. Effect of Mito-TEMPO on APAP+Fe^2+^-induced liver injury

**Fig. 1.**
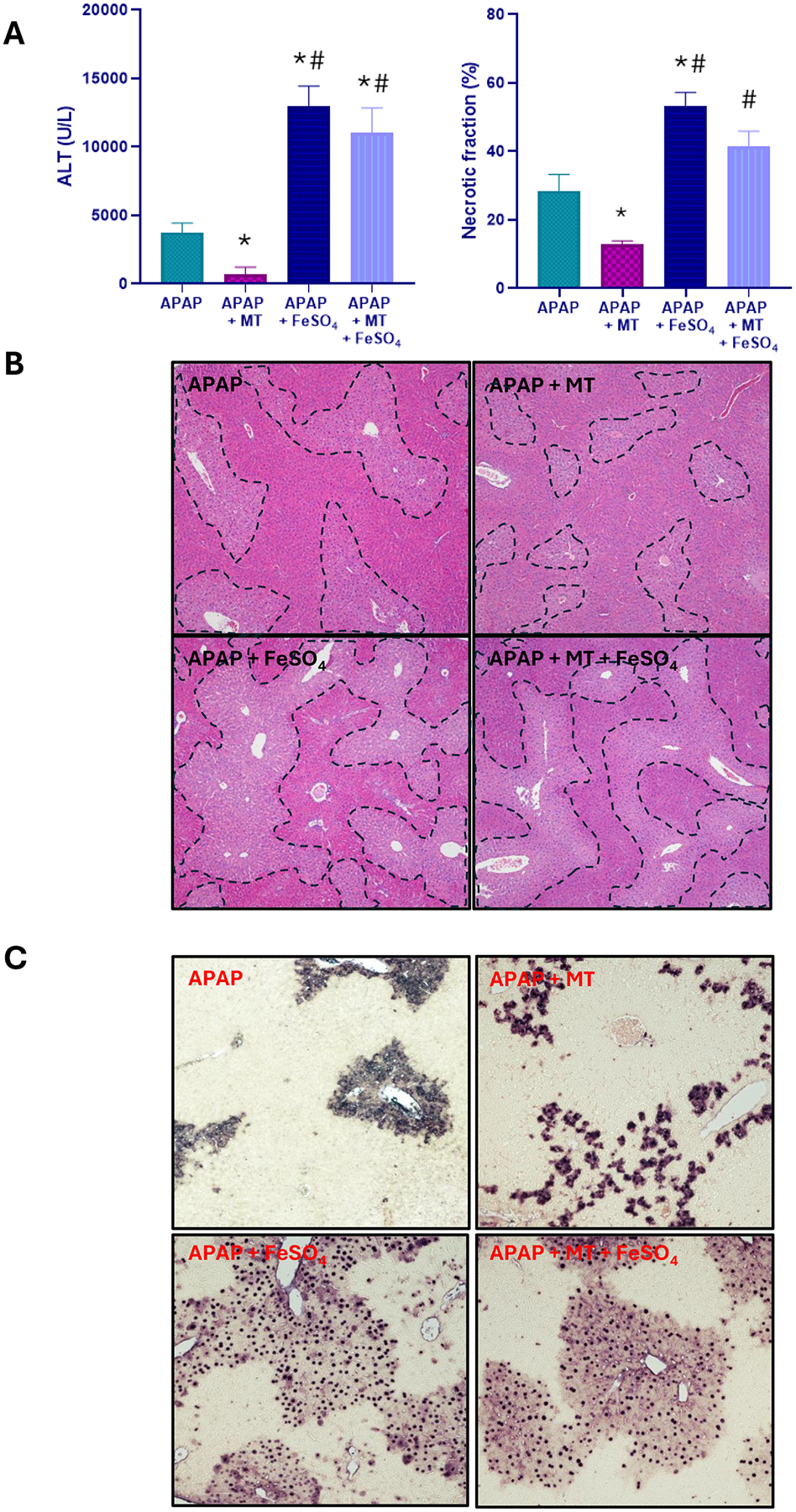
Effect of Mito-TEMPO on APAP+Fe^2+^-induced liver injury. Overnight fasted mice were treated with 300 mg/kg APAP with or without 0.15 mmol/kg FeSO_4_ 90 min before 20 mg/kg Mito-TEMPO. (A) plasma alanine aminotransferase (ALT) activity and necrotic area quantification, (B) representative H&E images showing areas of necrosis, and (C) representative TUNEL staining images 6 h after APAP overdose. Bars represent mean ± SEM for n = 4 mice per group. *P < 0.05 vs APAP; ^#^P < 0.05 vs APAP + MT.

**Fig. 2.**
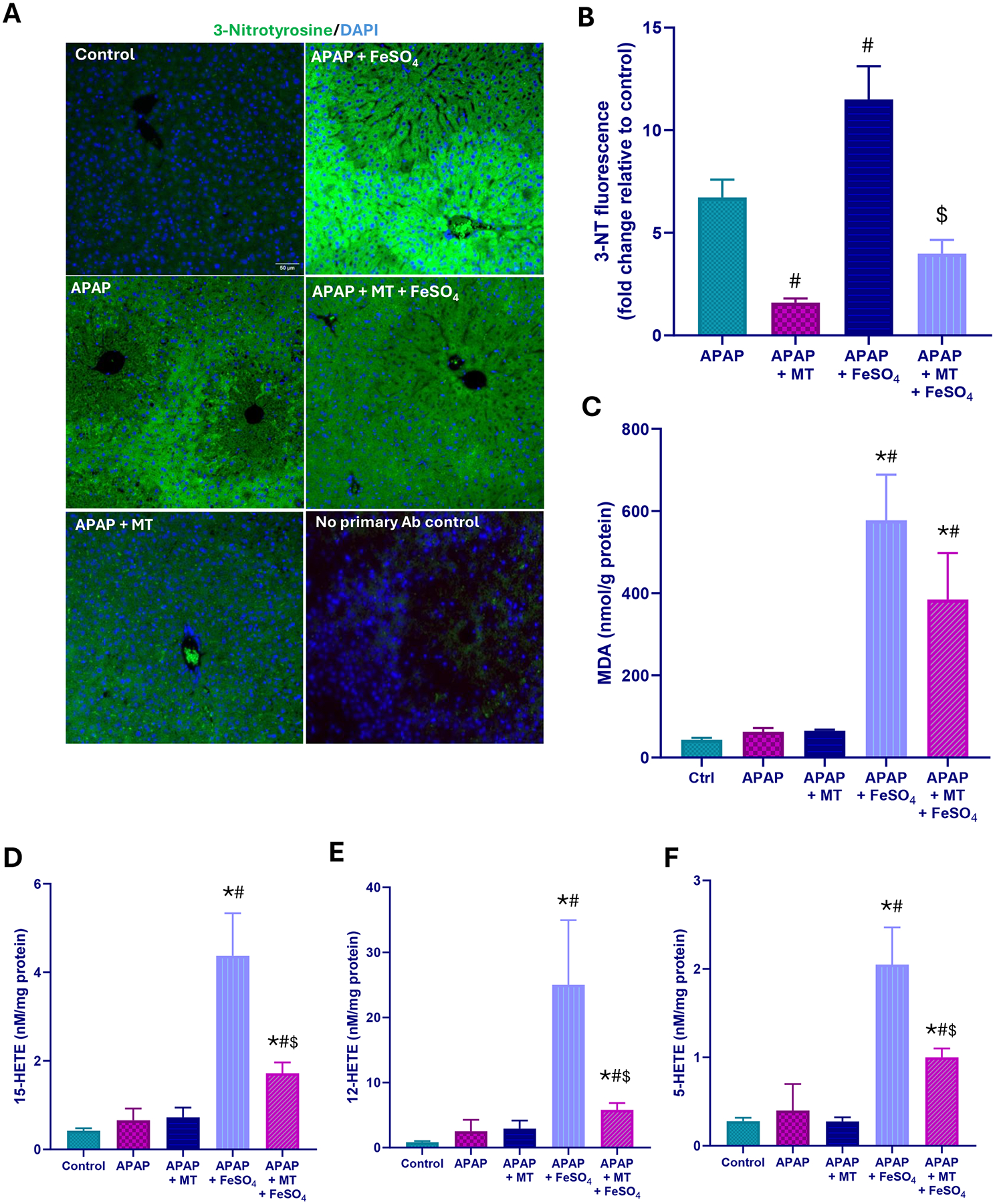
Effect of Mito-TEMPO on protein nitration and lipid peroxidation after APAP+Fe^2+^ co-treatment. (A) representative immunofluorescence images for 3-nitrotyrosine; a representative negative control (staining without the primary antibody) is shown from an APAP-treated animal with severe necrosis (B) quantification of 3-nitrotyrosine fluorescence (C) hepatic MDA (D) hepatic 15-HETE (E) hepatic 12-HETE, and (F) hepatic 5-HETE levels 6 h after APAP overdose. Bars represent mean ± SEM for n = 4–5 mice per group. *P < 0.05 vs control; ^#^P < 0.05 vs APAP; ^&^P < 0.05 vs APAP + MT; ^$^P < 0.05 vs APAP + FeSO_4_.

**Fig. 3.**
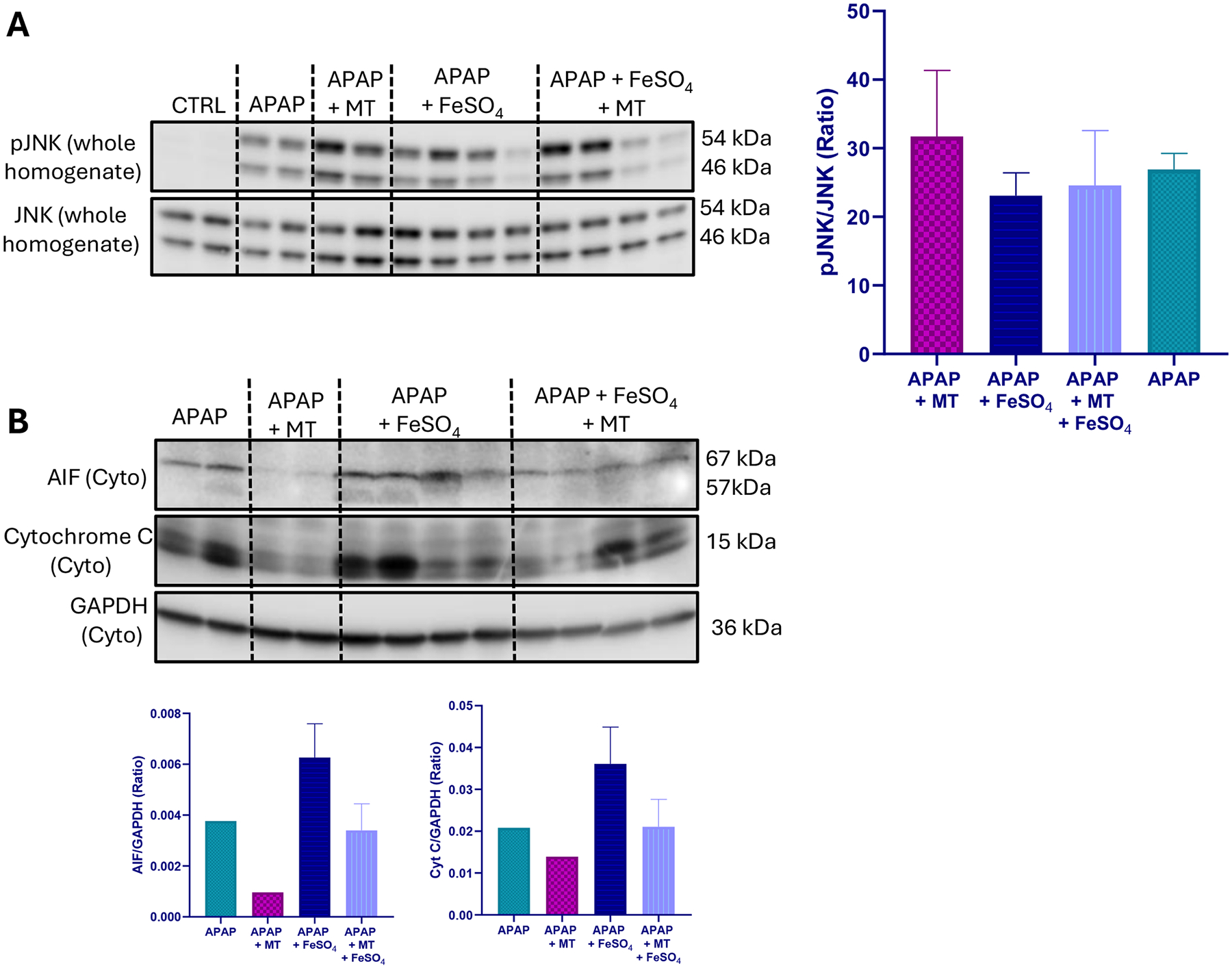
Effect of Mito-TEMPO on mitochondrial dysfunction after APAP+Fe^2+^ co-treatment. Western blot images and quantification showing (A) JNK activation and (B) cytosolic AIF and cytochrome c release 6 h after APAP overdose. Bars represent mean for n = 2 mice per group (Control, APAP, APAP+MT) and mean ± SEM for n = 4 animals per group (APAP+Fe^**2+**^, APAP+Fe^**2+**^+MT).

A moderate overdose of APAP (300 mg/kg) caused significant liver injury in fasted C57BL/6 J mice as indicated by the elevated plasma ALT activity, centrilobular necrosis, and DNA fragmentation (TUNEL assay) at 6 h after APAP (Fig. 1). The injury involved nitrotyrosine generation as seen on immunostaining, suggesting peroxynitrite formation, but no change in hepatic MDA levels measured as a parameter for LPO (Fig. 2A–C). Several hydroxy eicosatetraenoic acid (HETE) species were also assessed as indicators for GPx4 activity and LPO (Mathews et al., 1994). Hepatic levels of 5-, 12-, and 15-HETE were not significantly elevated after APAP compared to controls (Fig. 2D–F). However, there was JNK activation (Fig. 3A) and release of mitochondrial intermembrane protein such as AIF and cytochrome c into the cytosol suggesting mitochondrial dysfunction (Fig. 3B). Consistent with our previous report (Du et al., 2017), when treating the animals with the mitochondria-targeted SOD mimetic Mito-TEMPO 1.5 h after APAP, all aspects of the injury (ALT, necrosis, TUNEL, mitochondrial dysfunction) (Figs. 1, 3) and nitrotyrosine staining (Fig. 2A, B) were substantially reduced except for the MDA levels, which were not altered with APAP alone in any case (Fig. 2C). Similarly, the low hepatic HETE levels were also not significantly affected by Mito-TEMPO treatment (Fig. 2D–F). Thus, the accelerated removal of superoxide with Mito-TEMPO largely prevents peroxynitrite formation, reiterating that protein nitration in mitochondria and not LPO is the critical event causing APAP-induced cell necrosis.

When animals were co-treated with ferrous iron and APAP, all injury parameters were significantly further increased compared to APAP alone (Figs. 1–3). Importantly, MDA levels were now increased by > 10-fold over baseline values, indicating that relevant LPO after an APAP overdose was only observed in the presence of ferrous iron (Fig. 2C). Consistent with this finding, hepatic HETEs levels were significantly increased by 4-fold (5-HETEs), 11-fold (12-HETEs), and 5-fold (15-HETEs) (Fig. 2D–F). Another interesting observation was the TUNEL assay, which now shows more pronounced nuclear staining and less staining in the cytosol compared to the APAP and APAP+MT groups (Fig. 1C). To evaluate whether Mito-TEMPO is still protective under these conditions, liver injury and the hepatic content of MDA and HETEs were measured. Despite the profound protection of Mito-TEMPO against APAP toxicity, in the presence of additional iron, there was no protection based on plasma ALT activities, necrosis and TUNEL assay (Fig. 1). Furthermore, there was no significant reduction in MDA levels and nitrotyrosine staining was only marginally affected (Fig. 2A–C). In contrast to MDA, hepatic HETE levels were significantly reduced between 40 % and 70 % in the Mito-TEMPO-treated animals but the levels were still substantially higher than in animals treated with APAP alone (Fig. 2D–F). Interestingly, release of AIF and cytochrome c was reduced to the levels observed with APAP alone (Fig. 3). Together these findings indicate that when animals were co-treated with ferrous iron, substantial LPO developed, which now is at least an important if not the dominant injury mechanisms after APAP. This could imply that in the presence of some excess iron, the injury mechanism switches from a mitochondrial dysfunction-mediated necrotic process to a mechanism resembling ferroptosis, albeit with mitochondrial damage.

### 3.2. Minocycline and APAP+Fe^2+^-mediated liver injury

**Fig. 4.**
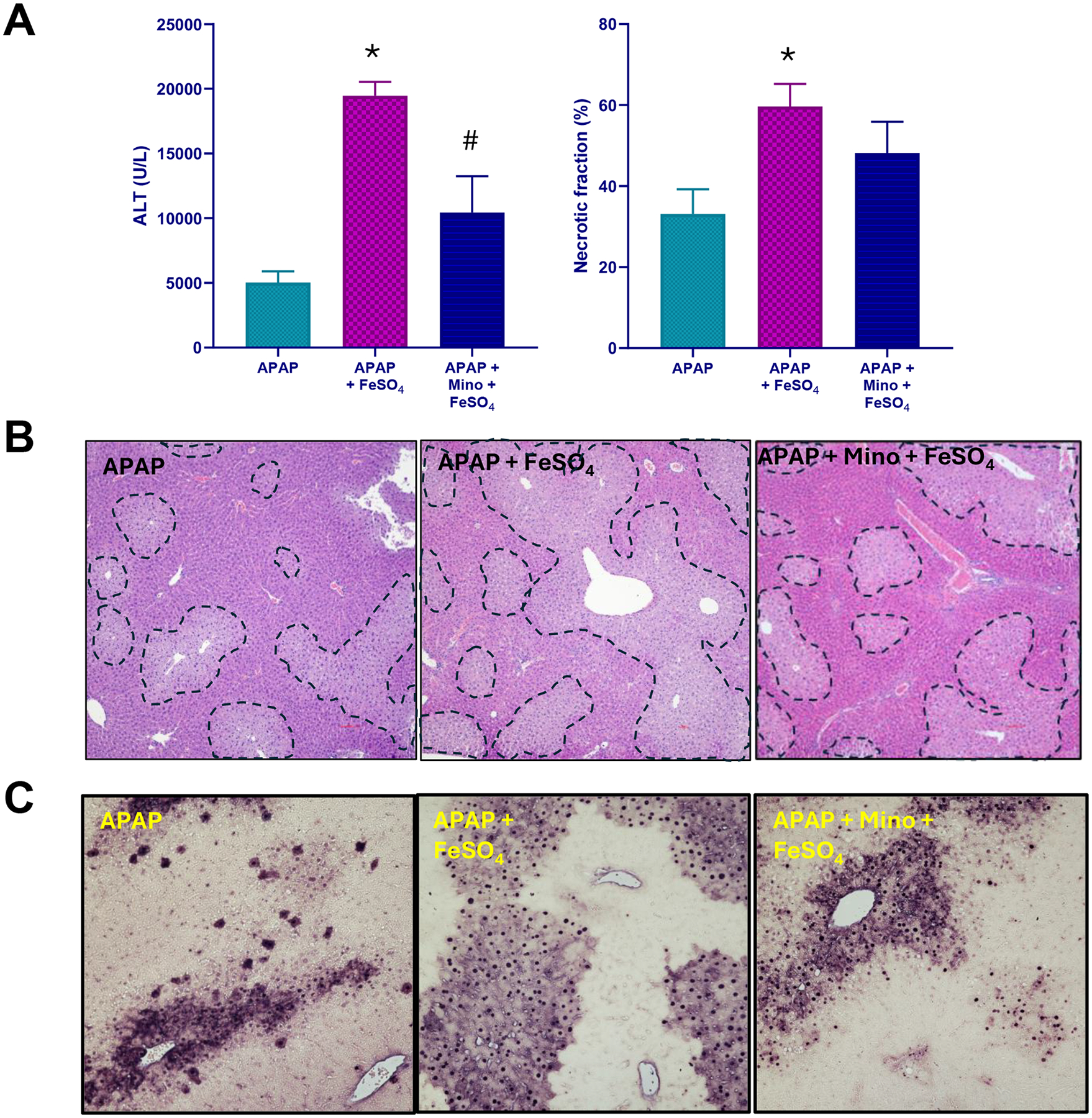
Effect of minocycline on APAP+Fe^2+^-induced liver injury. Overnight fasted mice were treated with 300 mg/kg APAP, or 300 mg/kg APAP + 0.15 mmol/kg FeSO_4_ with or without 1 h pretreatment with 10 mg/kg minocycline. (A) Plasma (ALT) activity and necrotic area quantification, (B) representative H&E images showing areas of necrosis, and (C) representative TUNEL staining images 6 h after APAP overdose. Bars represent mean ± SEM for n = 4 mice per group. *P < 0.05 vs APAP; ^#^P < 0.05 vs APAP + FeSO_4_.

**Fig. 5.**
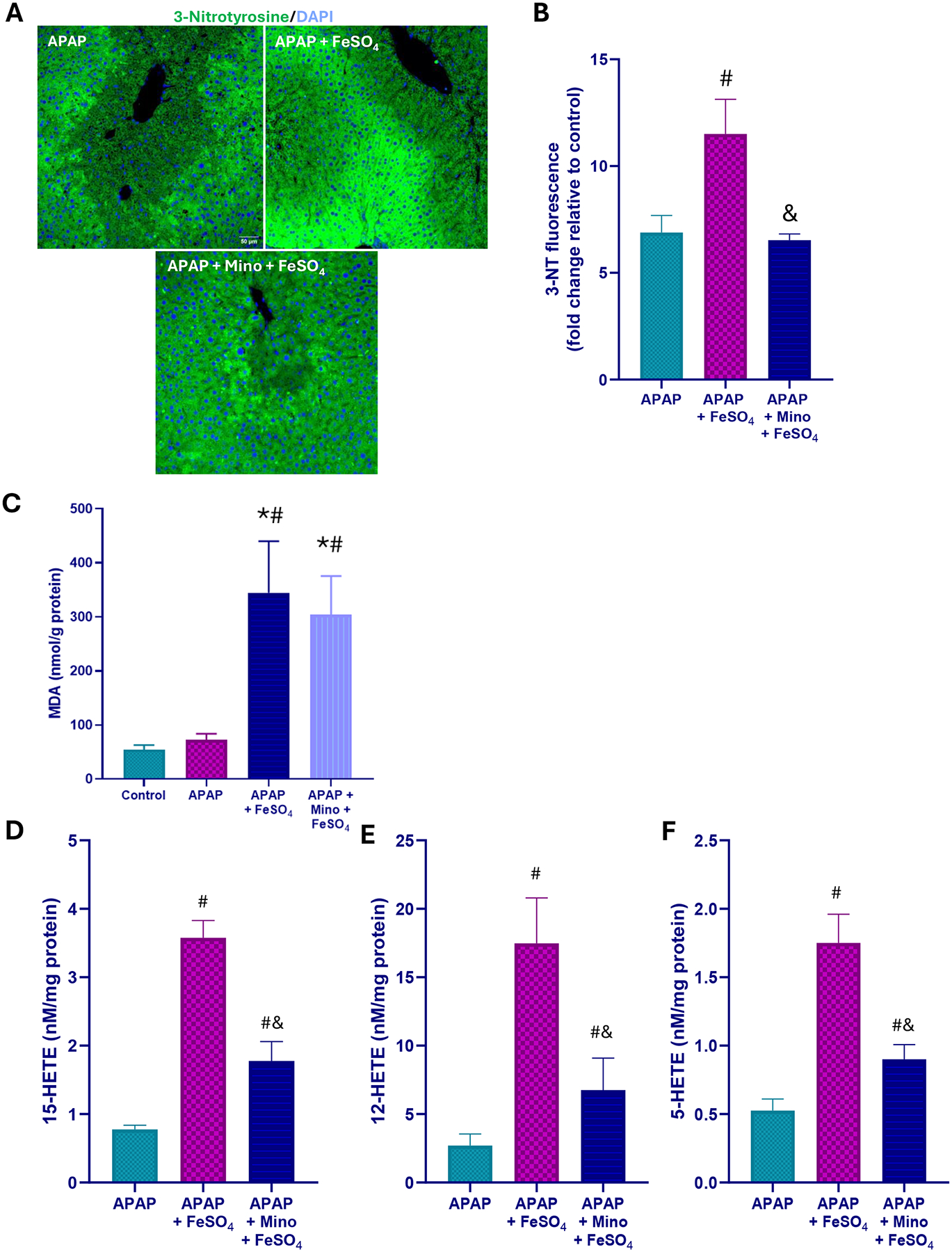
Effect of minocycline on protein nitration and lipid peroxidation after APAP+Fe^2+^ co-treatment. (A) representative immunofluorescence images for 3-nitrotyrosine, (B) quantification of 3-nitrotyrosine fluorescence, (C) hepatic MDA content, and (D) hepatic 15-HETE (E) hepatic 12-HETE, and (F) hepatic 5-HETE levels 6 h after APAP overdose. Bars represent mean ± SEM for n = 4–5 mice per group. *P < 0.05 vs control; ^#^P < 0.05 vs APAP; ^&^P < 0.05 vs APAP+FeSO_4_.

**Fig. 6.**
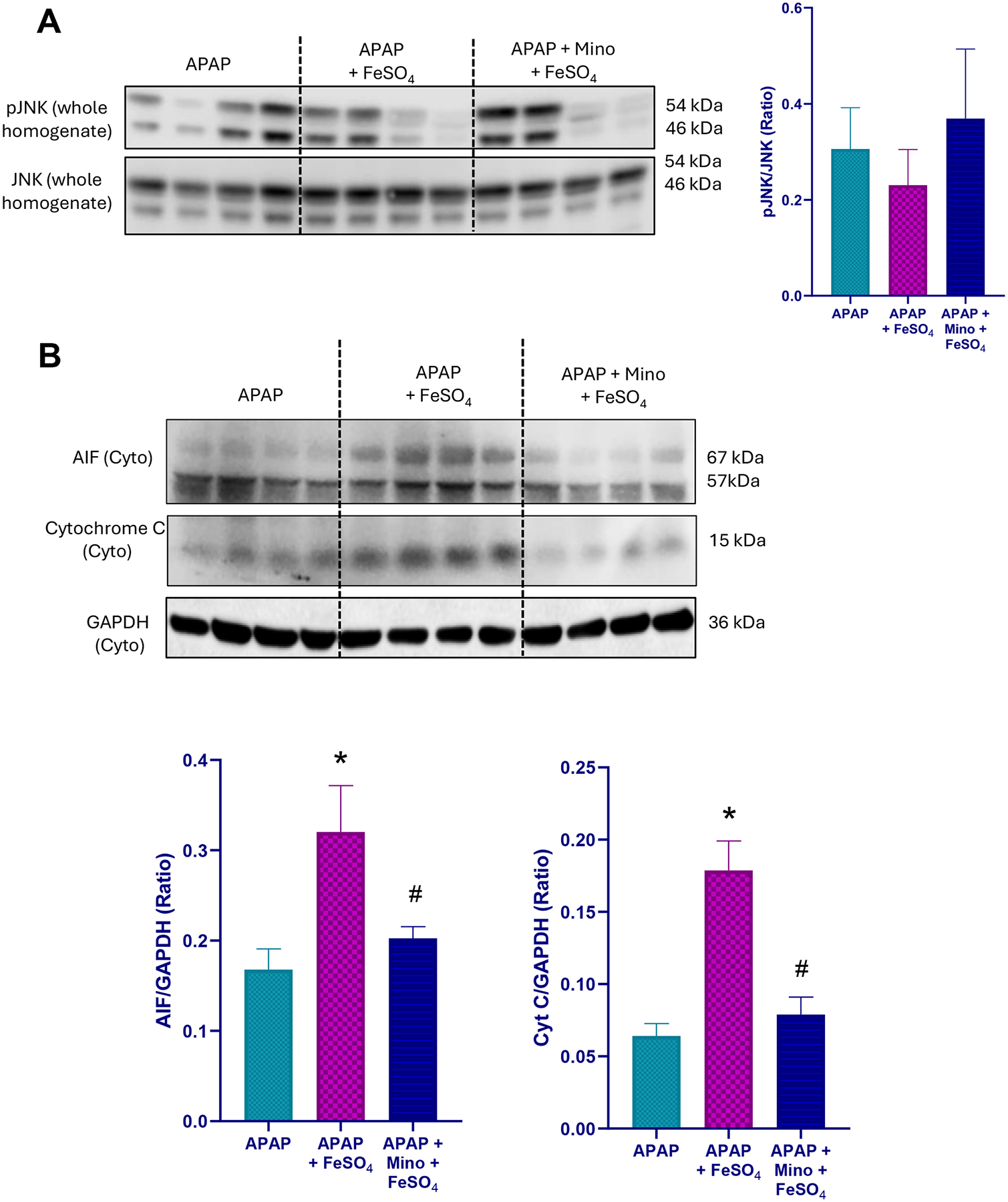
Effect of minocycline on mitochondrial dysfunction after APAP+Fe^2+^ co-treatment. Western blot images and quantification showing (A) JNK activation and (B) cytosolic AIF and cytochrome c release 6 h after APAP overdose. Bars represent mean ± SEM for n = 4 mice per group. *P < 0.05 vs APAP; ^#^P < 0.05 vs APAP+FeSO_4_.

Previous studies showed that minocycline, an inhibitor of the mitochondrial Ca^2+^ uniporter, can inhibit the transport of lysosomal ferrous iron into mitochondria and protect against APAP-induced liver injury in vitro and in vivo (Hu et al., 2016; Hu and Lemasters, 2020). In addition, minocycline reduced protein nitration after APAP alone (Adelusi et al., 2022). Therefore, it was tested whether pretreatment with minocycline also protects in the APAP+Fe^2+^ model. Minocycline moderately attenuated the increased injury based on a 46 % reduction in ALT activities and a trend to reduced necrosis and number of TUNEL-positive cells (Fig. 4). However, the hepatic MDA levels were similar (Fig. 5C) but nitrotyrosine staining was also partially reduced (Fig. 5A,B). In addition, hepatic levels of 5-HETE (−47 %), 12-HETE (−64 %) and 15-HETE (−44 %) were significantly reduced (Fig. 5D–F). Moreover, mitochondrial AIF and cytochrome c release was significantly attenuated (Fig. 6). Together, these findings suggest that mitochondrial iron uptake and protein nitration may still play a role, albeit a moderate one, in the pathophysiology of APAP+Fe^2+^-induced liver injury.

### 3.3. Are the standard antidotes NAC and fomepizole (4MP) still effective in the APAP+Fe^2+^ model?

**Fig. 7.**
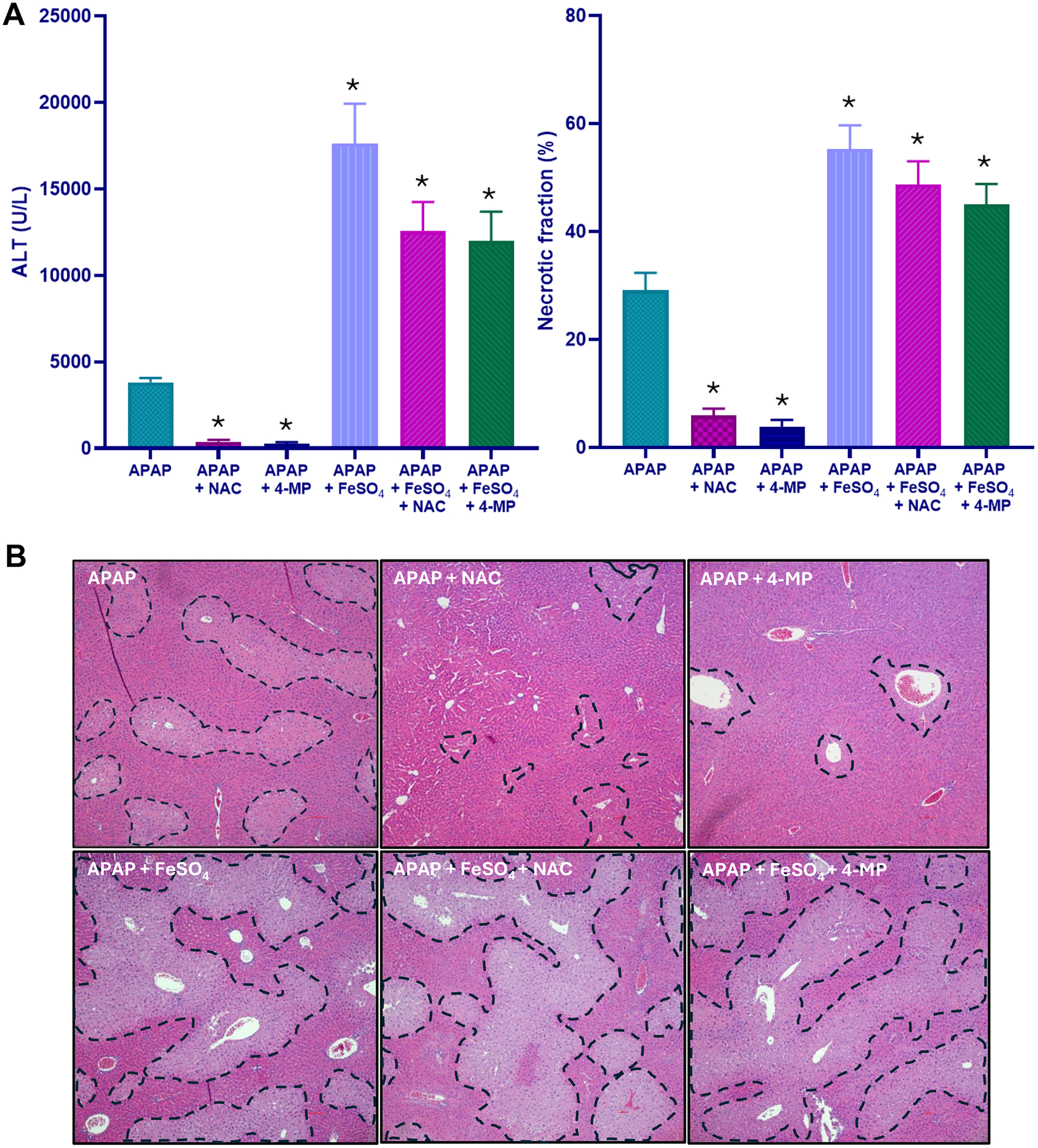
Effect of NAC and 4-MP on APAP+Fe^2+^-induced liver injury. Overnight fasted mice were treated with 300 mg/kg APAP, or 300 mg/kg APAP + 0.15 mmol/kg FeSO_4_ with or without 90-minute post-treatment with either 500 mg/kg NAC or 50 mg/kg 4-MP. (A) Plasma (ALT) activity and necrotic area quantification, and (B) representative H&E images showing areas of necrosis 6 h after APAP overdose. Bars represent mean ± SEM for n = 5 mice per group. *P < 0.05 vs APAP.

**Fig. 8.**
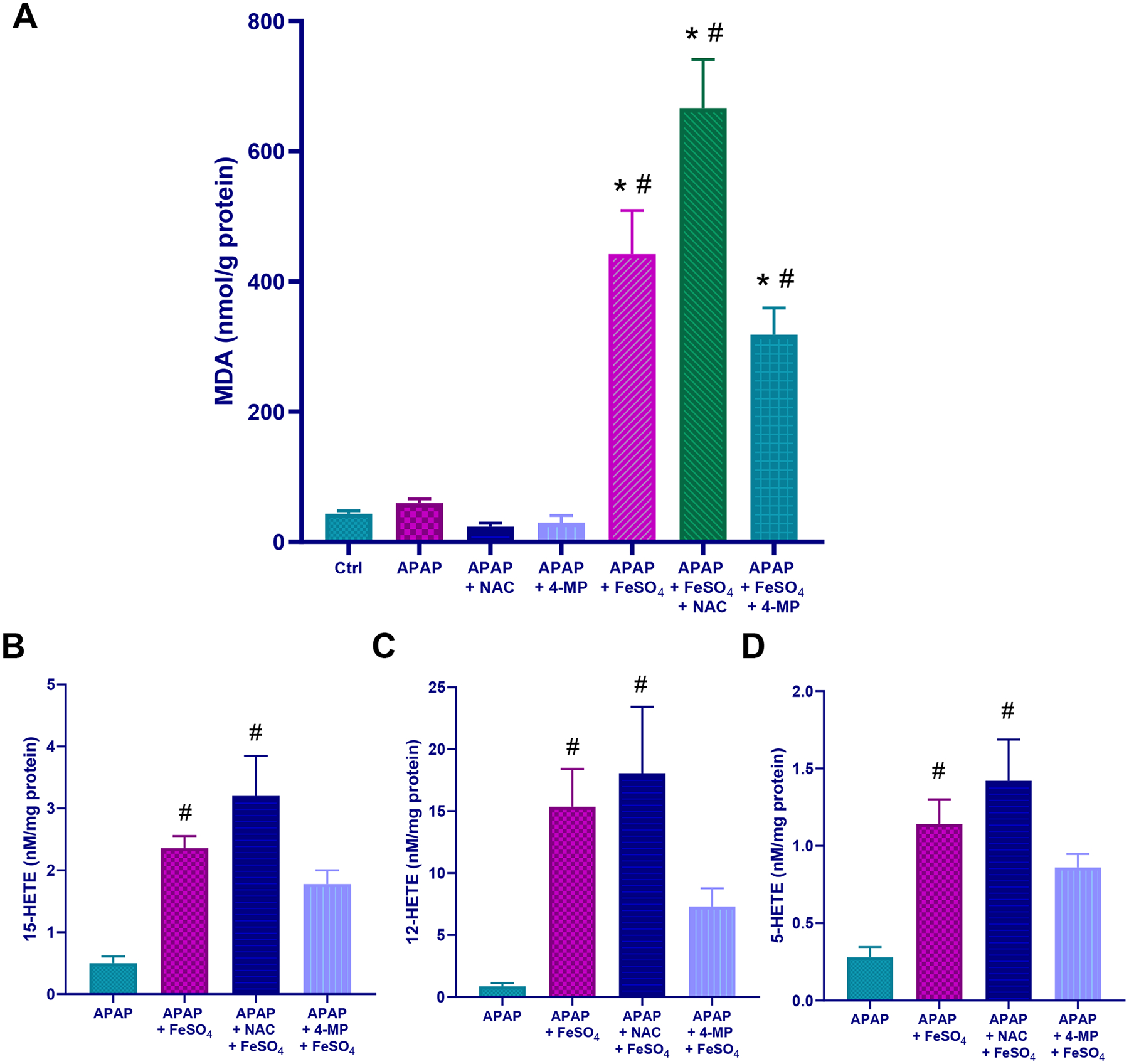
Effect of NAC and 4-MP on lipid peroxidation after APAP+Fe^2+^ co-treatment. (A) hepatic MDA content, and (B) hepatic 15-HETE (C) hepatic 12-HETE, and (D) hepatic 5-HETE levels 6 h after APAP overdose. Bars represent mean ± SEM for n = 5 mice per group. *P < 0.05 vs control; ^#^P < 0.05 vs APAP.

Our results so far indicated that the addition of a limited dose of exogenous ferrous iron can shift the standard mechanism of APAP-induced cell death towards a ferroptosis-like cell death mechanism. This raises the question whether the standard antidotes against APAP are still effective when administered after the metabolism phase. Both NAC and fomepizole (4MP) are known to almost completely protect against APAP hepatotoxicity when injected at 1.5 h after APAP in the mouse model (Akakpo et al., 2019; James et al., 2003; Knight et al., 2002; Saito et al., 2010). These observations could be confirmed in the current experiment. The substantial injury induced by APAP alone was reduced by 90 % (NAC) and 93 % (fomepizole) based on plasma ALT activities and by 80 % (NAC) and 87 % (fomepizole) based on the quantification of the areas of necrosis (Fig. 7A, B). In striking contrast to these findings, the identical treatment with NAC or fomepizole showed only a minor, non-significant reduction of plasma ALT activities and the areas of necrosis in animals exposed to APAP and Fe^2+^ (Fig. 7A, B). After treatment with APAP alone, MDA levels remained at baseline levels, and these numbers did not significantly change with either NAC or fomepizole treatment (Fig. 8A). However, after APAP+Fe^2+^, MDA levels, which were again dramatically increased in the Fe^2+^-treated group, were further elevated by 51 % (P > 0.05) after NAC treatment and reduced by 28 % (P > 0.05) in the fomepizole (4MP) group (Fig. 8A). NAC treatment did not affect the high hepatic HETE levels, but treatment with fomepizole (4MP) reduced 5-HETE (−47 %), 12-HETE (−62 %), and 15-HETE (−47 %) compared to APAP+Fe^2+^ (Fig. 8B–D).

## 4. Discussion

### 4.1. Role of LPO in APAP-induced liver injury

The objective of this investigation was to evaluate whether the co-treatment of APAP with ferrous iron shifts the mechanism of cell death towards LPO and ferroptosis-like mechanisms. We confirmed our previous reports that under normal conditions (APAP overdose alone), hepatotoxicity does not involve relevant LPO (MDA, 4-hydroxynonenal and HETEs) (Knight et al., 2003; Adelusi et al., 2022, 2024) but is characterized by extensive protein nitration (Knight et al., 2002, 2003; Cover et al., 2005), which could be eliminated by accelerated removal of superoxide through mitochondrial-targeted SOD mimetics (Du et al., 2017, 2019; He et al., 2023), iron chelation (Adelusi et al., 2022) and prevention of the uptake of endogenous ferrous iron into mitochondria with inhibition of the MPTP opening and cell death (Kon et al., 2010; Hu et al., 2016, 2023; Hu and Lemasters, 2020). These findings suggest that lysosomal-derived ferrous iron is a critical facilitator for peroxynitrite-mediated protein nitration in the mitochondria and important for the mitochondria-dependent cell death mechanism after an APAP overdose. This type of programmed necrosis, despite the critical role of GSH depletion, ROS, peroxynitrite and iron, lacks the involvement of LPO as a final cell death mechanism to be called ferroptosis (Iorga and Dara, 2019; Jaeschke et al., 2019; Jaeschke and Ramachandran, 2024b).

In contrast to normal conditions, co-treatment of APAP with ferrous iron did not only enhance protein nitration but now also caused extensive LPO (Adelusi et al., 2022). However, it remained unclear if LPO became a relevant contributor to cell death under these conditions. In striking contrast to APAP alone, delayed treatment with Mito-TEMPO did not show significant protection in the APAP+Fe^2+^ group when plasma ALT activities, areas of necrosis and TUNEL-positive cells were evaluated. In addition, MDA levels were not significantly different and hepatic HETE levels were only partially reduced. However, the release of mitochondrial intermembrane proteins was attenuated to levels observed with APAP alone and protein nitration was only moderately reduced. These observations suggest that there is some attenuation of mitochondrial dysfunction, which however has no relevant effect on the injury when ferrous iron is present with APAP overdose. The presence of protein nitration under these conditions may seem puzzling. However, protein nitration by peroxynitrite is a 2-step process (Campolo et al., 2014). The first step is the formation of a tyrosyl radical by reduction of the oxo-metal complex or the NO_2_ radical, which are decomposition products of peroxynitrite (Campolo et al., 2014). The second step is the combination of the tyrosyl radical with the NO_2_ radical, which yields 3-nitrotyrosine (Campolo et al., 2014). Under conditions of LPO, LO^.^ And LOO^.^ radicals can catalyze the first step and make the process more efficient requiring less peroxynitrite for protein nitration. Thus, the SOD mimetic, despite reduced overall peroxynitrite formation, has a more limited effect on protein nitration. Nevertheless, the results suggest that in the APAP+Fe^2+^ model, LPO plays a significant role as cell death mechanism indicating a shift towards ferroptosis-like cell death. It is important to recognize that the original definition of ferroptotic cell death induced by erastin in NRAS-mutant HT-1080 fibrosarcoma cells involved iron-induced LPO in the absence of mitochondrial changes (Dixon et al., 2012). However, although the current cell death induced by APAP+Fe^2+^ is also caused by iron-dependent LPO, it additionally involves substantial mitochondrial dysfunction. Thus, many aspects of this cell death are similar to the original ferroptosis but there are also clear differences. It is therefore more accurate to define this cell death induced by APAP+Fe^2+^ as “ferroptosis-like” cell death.

Another feature that shows the different mode of cell death in this model is supported by the TUNEL staining, which changes from total cellular staining with APAP alone to a more nuclear staining pattern in the presence of APAP+Fe^2+^. The nuclear DNA fragmentation is completely dependent on mitochondrial dysfunction with endonuclease G and AIF release and their translocation to the nucleus after APAP alone (Bajt et al., 2006; Cover et al., 2005). The altered nuclear staining pattern in the presence of iron, together with the reduced mitochondrial release of intermembrane proteins would suggest an attenuation but not absence of mitochondrial dysfunction. However, the fact that LPO and the injury is not reduced, supports the hypothesis that LPO is now a dominant injury mechanism, which is consistent with a ferroptosis-like mechanism of cell death.

The experiments with minocycline, an inhibitor of the mitochondrial Ca^2+^ uniporter, which prevents uptake of iron into the mitochondria (Hu et al., 2016), confirmed the observations with Mito-TEMPO and the respective conclusions. Although there was a reduction of intermembrane protein release and some reduction in protein nitration, i.e. some attenuation of mitochondrial dysfunction, no effect on MDA levels, a partial reduction in hepatic HETE content and only a very moderate overall effect on the injury (significant reduction in plasma ALT activities but only a trend in reduced necrosis) was observed. Together these results also suggest a more limited impact of protein nitration on the injury and a more dominant effect of LPO in this model when iron is co-administered with an APAP overdose. The fact that a general iron chelator eliminated LPO and the injury (Adelusi et al., 2022) but inhibiting mitochondrial uptake of iron did not significantly reduce LPO (MDA), and only modestly affected the injury suggests that under conditions of exogenous iron supply most of the critical LPO mainly takes place outside the mitochondria.

MDA is a product of LPO and HETEs are reduction products of lipid hydroperoxides, which are intermediate metabolites of LPO. HETEs are generated by GPx4 using GSH as electron donor. Thus, both MDA and HETEs can be considered indicators of LPO. Consistent with this conclusion, low levels of MDA and HETEs were measured in controls and after APAP overdose alone but a dramatic increase of all parameters was observed in the APAP+Fe^2+^ group indicating that based on both types of metabolites, relevant LPO occurs only in the ferrous iron-treated animals. It also suggests that despite aggressive progression of LPO and liver injury under these conditions, GPx4 is still active and is reducing lipid hydroperoxides. An interesting observation is that with Mito-Tempo and with minocycline, the MDA content was unaffected, but HETE levels were partially reduced. This may be caused by the fact that GPx4 activity can be affected not only by the accessible concentrations of lipid hydroperoxides but also by the co-factor GSH. In addition, the measured 3 HETEs are a selection of many possible metabolites of arachidonic acid and docosahexaenoic acid, the most susceptible fatty acid targeted during LPO (Jaeschke et al., 1987; Yamada et al., 2020). These issues need to be considered when interpreting the data. Nevertheless, HETEs are highly specific parameters of LPO.

### 4.2. How clinically relevant is the APAP+Fe^2+^ model?

If a manipulation of the experimental conditions shifts the mode of cell death, the obvious question is about the clinical relevance of the model. Vitamin E deficiency with enhancement of PUFAs in the cell membrane dramatically increased the susceptibility to APAP hepatotoxicity and LPO (Wendel et al., 1979; Wendel & Feuerstein, 1981). However, vitamin E deficiency in adults and older children is correlated with food insecurity mainly in developing countries (Dror and Allen, 2011) where ALF due to APAP overdose is limited. In Western countries with more access to APAP, where an overdose is the prominent cause of ALF, vitamin E deficiency is rare and related to fat malabsorption disorders or voluntary intake of very low-fat diets (Traber and Sies, 1996), which also reduces the levels of PUFAs in the membranes. Vitamin E deficiency with elevated levels of PUFAs and APAP overdoses is an extremely rare combination in humans and thus the relevance for APAP-induced ferroptosis-like cell death is negligible. However, the ferrous iron treatment used in the current study is more realistic. First, the dose of 0.15 mmol/kg in ferrous sulfate (22.8 mg/kg body weight) represents a human equivalent dose of 1.9 mg/kg body weight. Second, a single dose of an iron supplement (325 mg per tablet) is approximately 5.4 mg/kg for a person with 60 kg body weight, which means that the iron sulfate dose in the animal experiments is well below doses used chronically in humans. Does that mean that taking iron supplements enhances the risk for APAP hepatotoxicity? This appears unlikely as the additionally supplied ferrous iron is rapidly incorporated into heme proteins or stored in ferritin. The danger of iron supplements comes from the co-administration of iron with an APAP *overdose*. Thus, if iron supplement tablets are being consumed with an APAP overdose during a suicide attempt (Audimoolam et al., 2011; Nye and Singh, 2022), a severe aggravation of the injury due to the initiation of LPO can occur in these patients. Thus, unless the patient overdoses on APAP while taking an iron supplement, the described mechanisms will not be induced.

### 4.3. Do regular antidotes still protect against APAP+Fe^2+^ toxicity?

Given the danger of LPO-induced liver injury, this also raises the question whether APAP+Fe^2+^ toxicity requires additional therapeutic strategies beyond the clinically approved antidote NAC and the antidote under clinical development fomepizole (Ramachandran et al., 2024). Previous studies have shown that delayed treatment with NAC, GSH or fomepizole still effectively protects against APAP-induced liver injury (Akakpo et al., 2019; Cover et al., 2005; James et al., 2003; Knight et al., 2002; Saito et al., 2010). For GSH and NAC given 1.5 h after APAP, i.e. at a time where the metabolic activation (NAPQI formation) is over after a dose of 300 mg/kg (Akakpo et al., 2019), the newly synthesized GSH scavenges peroxynitrite in mitochondria and prevents cell necrosis (Knight et al., 2002). On the other hand, fomepizole treatment 1.5 h after APAP inhibits JNK activation and prevents the mitochondrial oxidant stress and cell death (Akakpo et al., 2019). In either case, the protective effect of these agents is close to 100 % even when given after the metabolism phase is over, which was again confirmed in the current experiments. In striking contrast to these many published experiments, the same or even higher doses of NAC given to animals treated with APAP+Fe^2+^ did not protect and did not reduce LPO. In fact, MDA and HETEs levels trended higher. Fomepizole treatment in these animals only mildly reduced LPO and showed only a trend of reduced injury. These results demonstrate that the very high efficacy of NAC or fomepizole in animals treated with a moderate APAP overdose, is greatly reduced under conditions when iron is present and LPO is the dominant mechanism of cell death. Given that the mouse model accurately reflects the human pathogenesis, (Ramachandran and Jaeschke, 2017), these data suggest that the clinically approved antidote NAC and the new antidote under clinical development fomepizole for APAP toxicity in patients may be much less effective in the presence of ferrous iron than after an APAP overdose alone. However, it needs to be kept in mind that early treatment during the metabolism phase, GSH synthesized by NAC (Corcoran et al., 1985; Knight et al., 2002) and the Cyp2E1 inhibition by fomepizole (Akakpo et al., 2018) will still reduce metabolic activation of APAP and thus injury even in patients overdosed on APAP+Fe^2+^. In contrast, the therapeutic window of these standard antidotes will be substantially reduced due to the initiation of massive LPO where NAC and fomepizole are no longer effective. Previous studies, at least in primary human hepatocytes, have indicated that fomepizole may have a larger therapeutic window than NAC (Akakpo et al., 2022). Although this may need to be confirmed in future studies, it also appears that fomepizole may be slightly more effective in reducing LPO and have at least a trend of protection in the APAP+Fe^2+^ model. This effect may be due to the higher efficiency of preventing NAPQI or ROS formation than scavenging these reactive metabolites with GSH. Nevertheless, under these more accelerated injury conditions driven by LPO, the clinically approved iron chelator deferoxamine mesylate may be the most effective adjunct treatment option (Adelusi et al., 2022). In addition, potent ferroptosis inhibitors such as liproxstatin-1 (Zilka et al., 2017) or UAMC-3203 (Devisscher et al., 2018) could be future adjunct treatment options. UAMC-3203 proved to be partially effective in the APAP+Fe^2+^ model (Adelusi et al., 2024).

## 5. Summary and conclusions

**Fig. 9.**
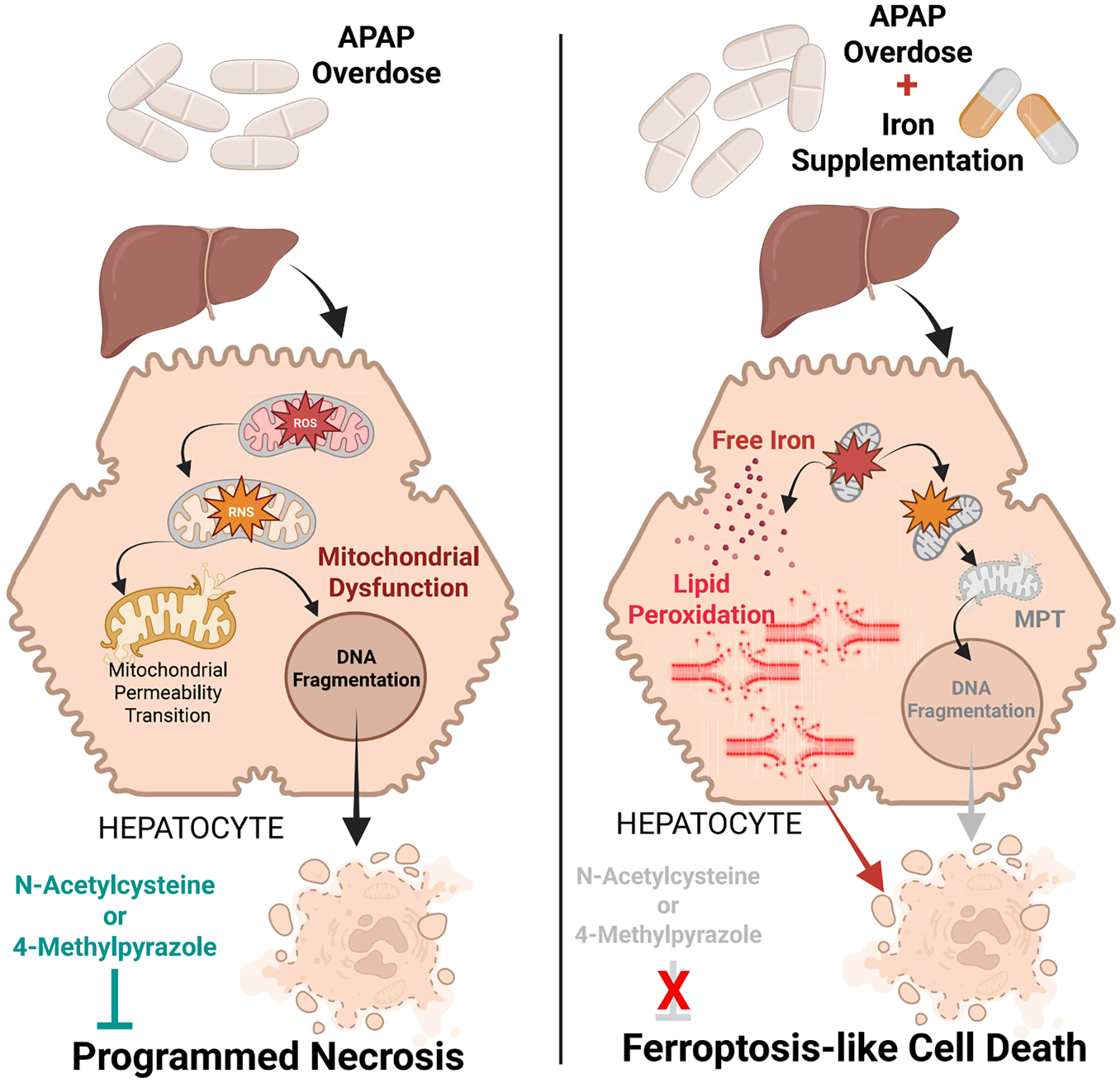
Switch in the mechanism of cell death when an APAP overdose is accompanied by iron supplementation. An APAP overdose typically induces mitochondrial dysfunction due to the formation of a reactive metabolite, which produces mitochondrial protein adducts. This causes the generation of reactive oxygen and nitrogen species within the organelle, inducing the mitochondrial permeability transition (MPT) pore opening and nuclear DNA fragmentation to trigger hepatocyte programmed necrosis, which can be prevented by N-Acetylcysteine (NAC) or 4-Methylpyrazole (4MP). However, when the APAP overdose is accompanied by iron supplementation, the free iron within hepatocytes facilitates substantial lipid peroxidation, which then induces ferroptosis-like cell death, where NAC and 4MP have limited benefits. (Created with Biorender.com).

An overdose of APAP causes severe liver injury mediated by mitochondrial oxidant stress, peroxynitrite formation, and mitochondrial dysfunction in the absence of relevant LPO in mice and humans. Endogenous ferrous iron mobilized from lysosomes facilitates mitochondrial protein nitration and the MPT pore opening (Fig. 9). In contrast, supplying moderate, non-toxic amounts of exogenous ferrous iron at the time of the APAP overdose causes a severe aggravation of liver injury with enhanced protein nitration but also with dramatically increased levels of LPO (Fig. 9). Based on the fact that interventions preventing protein nitration through reduction in peroxynitrite formation (Mito-Tempo) or blocking iron-mediated catalysis of protein nitration in mitochondria (minocycline) after APAP alone are largely ineffective in animals treated with APAP+Fe^2+^ (Fig. 9), it can be concluded that the mode of cell death shifts from the mitochondriacentric programmed necrosis to ferroptosis-like cell death with LPO as the dominant cell death mechanism, though the mode of cell death still differs from classical ferroptosis due to the concomitant mitochondrial dysfunction (Dixon et al., 2012). This conclusion regarding shift of cell death mechanism is supported by the fact that traditional antidotes, which protect after APAP alone by scavenging peroxynitrite in mitochondria (NAC, which synthesizes GSH) or by preventing the mitochondrial oxidant stress (fomepizole), are ineffective in the APAP+Fe^2+^ model when treated after the metabolism phase. Thus, LPO-mediated ferroptosis is irrelevant as cell death mechanism under normal conditions but a disturbance of this delicate redox-balance by co-ingestion of non-toxic amounts of ferrous iron together with an APAP overdose can trigger severe LPO and ferroptosis-like cell death. Although a rare occurrence in patients, it is important to recognize this problem as it requires additional interventions beyond the traditional antidotes, such as iron chelation.

## Data Availability

Data will be made available on request.

## Abbreviations:

AIF: apoptosis inducing factor
ALT: alanine aminotransferase
APAP: acetaminophen
Cyp2E1: cytochrome P450 2E1
GSH: glutathione
HETE: hydroxy eicosatetraenoic acid
H&E: hematoxylin & eosin
IHC: immunohistochemistry
JNK: c-Jun N-terminal kinase
LPO: lipid peroxidation
MDA: malondialdehyde
4MP: 4-methylpyrazole fomepizole
MPTP: mitochondrial permeability transition pore
NAC: N-acetyl-L-cysteine
NAPQI: N-acetyl-p-benzoquinone imine
ROS: reactive oxygen species
SOD: superoxide dismutase
TUNEL assay: terminal deoxynucleotidyl transferase dUTP nick-end labelling assay
